# Characterizing Trends of Lymphedema After Axillary Lymph Node Dissection with and Without Immediate Lymphatic Reconstruction

**DOI:** 10.3390/cancers17182964

**Published:** 2025-09-10

**Authors:** Kella L. Vangsness, Andre-Philippe Sam, Jeff Chang, Yash A. Mehta, Michael W. Chu, Mouchammed Agko, Antoine L. Carré

**Affiliations:** 1 Division of General Surgery, Community Memorial Hospital, Ventura, CA 93033, USA; 2 Division of Plastic Surgery, Department of Surgery, City of Hope, Duarte, CA 92618, USA; asam003@medsch.ucr.edu (A.-P.S.); jechang@coh.org (J.C.); yash.mehta@medsch.ucr.edu (Y.A.M.); magko@coh.org (M.A.); acarre@coh.org (A.L.C.); 3 Division of Plastic and Reconstructive Surgery, Kaiser Permanente, Los Angeles, CA 90027, USA; michael.w.chu@kp.org

**Keywords:** lymphedema, immediate lymphatic reconstruction, axillary lymph node dissection, LYMPHA

## Abstract

**Background and Objectives:** Breast cancer-related lymphedema (BCRL) is a complication of axillary lymph node dissection (ALND). Immediate lymphatic reconstruction (ILR) may help to decrease lymphedema after ALND by creating lymphatic bypasses. This retrospective single-institution study aimed to compare lymphedema in patients undergoing ALND with and without ILR. **Materials and Methods:** Bioimpedance and limb measurements determined the presence of BCRL. The categorical data that were collected and analyzed included BMI, comorbidities, BCRL onset, and number of lymphatic bypasses. Pearson’s chi-square test and multivariable logistic regression were performed to identify factors associated with the onset of lymphedema. An odds ratio compared the incidence of BCRL with and without ILR. **Results:** In total, 186 patients underwent ALND, 44 (24%) with ILR and 142 (76%) without. The mean number of bypasses during ILRs created was 3.54. The odds of developing lymphedema with ILR were 64% lower than for ALND alone. ILR patients who developed BCRL had a mean onset of 543 days post-operatively versus 389 days in the control group. Age, ethnicity, BMI, and bypass amount had no significant influence on lymphedema development. **Conclusions:** ILR was associated with lower rates of BCRL after ALND. Patients who developed lymphedema despite undergoing ILR did so 8 months later than the controls.

## Simple Summary

Breast cancer-related lymphedema is a complication following axillary lymph node dissection causing extensive morbidity and mortality. Current treatment options for lymphedema are limited. Immediate lymphatic reconstruction allows for the continued movement of lymph fluid. This is done by diverting it into veins through the creation of a lymphovenous bypass. The aim of this technique is to provide a substantial optios for patients undergoing axillary lymph node dissection.

## 1. Introduction

Breast cancer is the most diagnosed cancer amongst women globally [1] and axillary lymph node dissection (ALND) is used in both the treatment and staging of breast cancer [2]. Although ALND provides therapeutic and diagnostic benefit, it can lead to the development of lymphedema, specifically known as breast cancer-related lymphedema (BCRL). BCRL is the disruption of lymphatic vessels causing lymphatic fluid accumulation in the axilla and upper extremities. Symptoms include swelling, decreased mobility in the operative arm [3], decreased activities of daily living, and decreased self-esteem [4,5].

The lymphatic system is a vital network of vessels that hold major roles in fluid homeostasis and inflammatory response [6,7]. The disruption of the lymph system in an upper extremity following axillary lymph node dissection or radiation has been heavily linked to psychological distress, poor quality of life [8], pain and functional disability [9], and prolonged wound healing [4,6]. While cures for lymphedema remain elusive due to the iatrogenic nature of its development, it is vital to employ preventative strategies.

**Figure 1:**
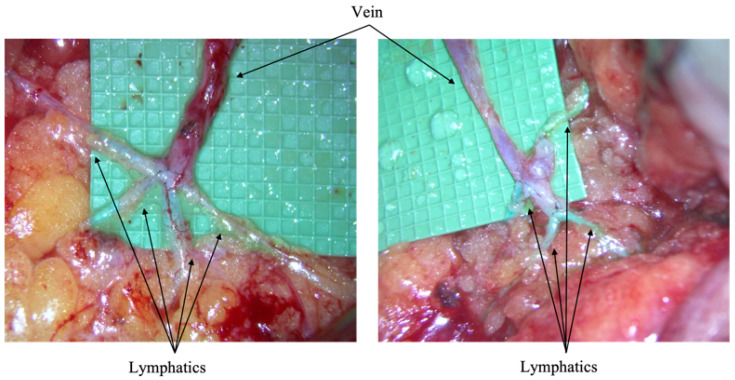
Above images feature examples of the LYMPHA procedure. A microsurgery bypass is created through the anastomosis of a vein (superiorly) and lymphatics (inferiorly) to allow for lymphatic flow. Intraoperative images taken from City of Hope, Duarte, California.

Reconstructive surgeons currently utilize immediate lymphatic reconstruction (ILR) to reduce the risk of postoperative lymphedema. ILR employs a lymphatic microsurgical preventative healing approach (LYMPHA), a microsurgical intervention that involves creating lymphovenous bypasses at the time of ALNDs [10,11,12]. The LYMPHA procedure creates an anastomotic bypass with a standard microsurgical technique between a lymphatic channel and vein (Figure 1) to allow for continued lymph flow and prevent localized pooling. Few studies have demonstrated promising results with ILR, yet they have limited cohort sizes and short follow-up periods [13,14]. The goal of developing ILR is to employ surgical techniques to decrease the incidence of lymphedema and improve post-operative outcomes. The aim of this study is to assess if ILR is an appropriate procedure for the prophylactic treatment of lymphedema in patients who have undergone axillary node dissections with and without ILR for the treatment of breast cancer.

### Immediate Lymphatic Reconstruction Technique

ILRs of lymphovenous anastomoses were performed intraoperatively following ALND at the time of the index surgery. A mixture of injectable isosulfan blue (IB) and flourescin isothiocyanate (FITC) dye was used to identify lymphatic vessels by injecting dye into the ipsilateral arm. Protocol for injection followed the Israel Deaconess Medical Center protocol [15]. Axillary veins were isolated and selected based on the desired length with minimal backflow. An ipsilateral dorsum foot vein was dissected for graft use if axillary veins were not sufficient in length. The locations of bypasses were determined by the amount and configuration of axillary lymphatic channels, as well as their proximity to venous tributaries. The maximum number of bypasses were sought for each procedure. Once created, intraoperative patency of lymphovenous anastomoses and flow of lymph fluid was evaluated through the combination IB and FITC dye under microscopic visualization.

## 2. Materials and Methods

This retrospective single-institution analysis was approved by the Institutional Review Board at City of Hope National Medical Center in Duarte, California (Protocol #23581, Reference #253089). Patients who underwent ALND with or without ILR between 2019 and 2023 were included. The primary outcome was the presence of lymphedema measured by SOZO (ImpediMed Limited, Brisbane, Australia). They were scored using the Lymphedema Index (L-Dex) (ImpediMed Limited, Brisbane, Australia) and upper limb circumference measurements performed by licensed lymphedema therapists. SOZO is a validated bioimpedance spectroscopy tool used for quantifying extracellular fluid shifts in lymphedema. It has high sensitivity and positive predictive value in BCRL with measurements scored using the L-Dex [16,17]. An increase of >6.5 units from baseline or >10 units from preoperative measurement is defined as subclinical lymphedema, while a score greater than +10 units is positive for lymphedema. Exclusion criteria included pre-existing lymphedema diagnoses, males, and patients who did not receive ALND.

The control group included patients who underwent ALND alone prior to 2020, as this was the year ILR was first available at the institution. The intervention group consisted of patients who were treated from 2020 onward and who underwent ALND with ILR. Clinical practices remained consistent during the study period without changes to assessment protocols. Two patients treated after 2020 received ALND only without ILR intervention due to intraoperative technical constraints, including insufficient lymphatic vessels and venous backflow. No additional patients were included or excluded based on intraoperative technical difficulties. Follow-up timelines were standardized across both groups to reduce time-related bias. The incidence and onset of lymphedema were recorded.

Patient data collected included age, race/ethnicity, body mass index (BMI), comorbidities, tumor stage, histologic grade, receptor subtype, the number of lymph nodes excised, and the number of lymphovenous bypasses created during ILR (Table 1). BMI was classified into underweight, normal, overweight, and obese, following World Health Organization criteria. For all patients, the mean BMI was 29 and age was 53. All patients received routine follow-up care and lymphedema screening approximately three months postoperatively. Assessments included a physical exam, limb measurement, and an L-Dex score.

### Statistical Analysis

ALND-only patients served as the control group while those who received ILR comprised the interventional group. Patient demographics and clinical characteristics were summarized with descriptive statistics. Categorical variables were compared using Pearson’s chi-squared test to identify associations. Despite data being sparsely reported or missing due to the nature of retrospective data collection, this test was employed across all categorical variables to maintain methodological uniformity. These results were assessed with caution given the known limitations of the chi-square test when expected cell counts are <5. Despite this analysis not revealing any significant relationship, we hypothesized that the number of positive lymph nodes removed might still be associated with the incidence of lymphedema when controlling for other factors. Continuous variables were analyzed for normality using appropriate central tendency and dispersion measures based on data distribution.

Assessment of ILR and associated lymphedema development was evaluated by calculating odds ratios and 95% confidence intervals. Multivariate logistic regression analyses using a binary logistic regression was used to assess for independent predictors of lymphedema. The model included BMI and age as continuous variables. Stages were separated into groups: stage 2 versus stage 3. Stage 2 acted as the reference category, since no patients were categorized as stages 0 or 1. Meanwhile, 0–9 +LN removed was the reference category for all +LN groups. Binary categories included extranodal extension, ILR, radiation, and adjuvant chemotherapy. Covariates included the number of lymph nodes removed and the number of lymphovenous bypasses performed. The numbers of positive lymph nodes (+LN) removed were separated into three categories: 0–9, 10–15, and 16+. A *p*-value < 0.05 was considered statistically significant.

## 3. Results

Out of the 186 patients examined, 142 (76%) received ALND only and 44 (24%) patients underwent ILR. Overall, only 52 (28%) patients developed lymphedema. When separated by control versus interventional groups, 44 of 142 (31%) ALND-only patients developed lymphedema whereas only 8 of 44 (18%) of those who underwent ILR developed the condition.

The mean age of patients in the ILR cohort was 44 years old versus 56 in the ALND only group. The race/ethnicity distribution of the entire cohort included 69 patients who identified as Hispanic/Latino, 65 as Caucasian/White, 38 as Asian/Pacific Islander, and 14 as Black/African American.

Descriptive analyses using a chi-squared test assessing differences in the number of +LN removed found no statistically significant findings, only observational differences, and none that were statistically significant. There were no patients in the 16+ +LN group who underwent ILR. However, 33% of patients in the 0–9 +LN group and 20% of the 10–15 +LN group underwent ILR (*X*^2^ (2, *n*= 212) = 3.63, *p* = 0.163). These findings are taken with a note of caution due to the small sample size, unequal group size, and cells which did not meet qualifying numbers for adequate analysis.

**Figure 2:**
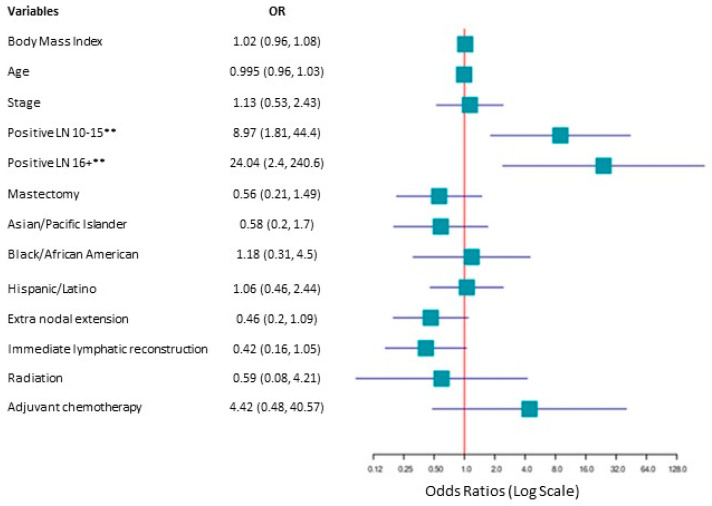
Odds ratio plot depicting demographic and clinical variables with the number of positive lymph nodes as a categorical variable. Body mass index and age were continuous variables. Stage compared stage 2 vs. stage 3; the reference category was stage 2. Positive lymph nodes with 0–9 +LN were used as a reference category. Mastectomy: yes vs. no, with no as a reference category. Race/ethnicity is presented with Non-Hispanic white as the reference category. Extranodal extension: no vs. yes, with no as a reference category. Immediate lymphatic reconstruction: no vs. yes, with no as a reference category. Radiation: no vs. yes, with no as the reference category. Adjuvant chemotherapy: no vs. yes, with no as the reference category. ** *p* < 0.01 was deemed statistically significant.

The results of the multivariable logistic regression assessing patient demographic or clinical variables found only one statistically significant relationship in the ILR group. This was between the number of positive lymph nodes removed when groups were analyzed as categorical variables (1–9, 10–15, 16+ positive lymph nodes) (Figure 2). Groups with 10–15 and 16+ +LNs removed had a 9 and 24 times increased likelihood of being diagnosed with lymphedema, respectively. These findings are taken with a note of caution due to the unequal group sizes and small sample size. Age, BMI, race/ethnicity, mastectomy, adjuvant chemo, radiation, ILR intervention, or extranodal extension were not significantly associated with the presence of lymphedema when examined as categorical variables.

**Figure 3:**
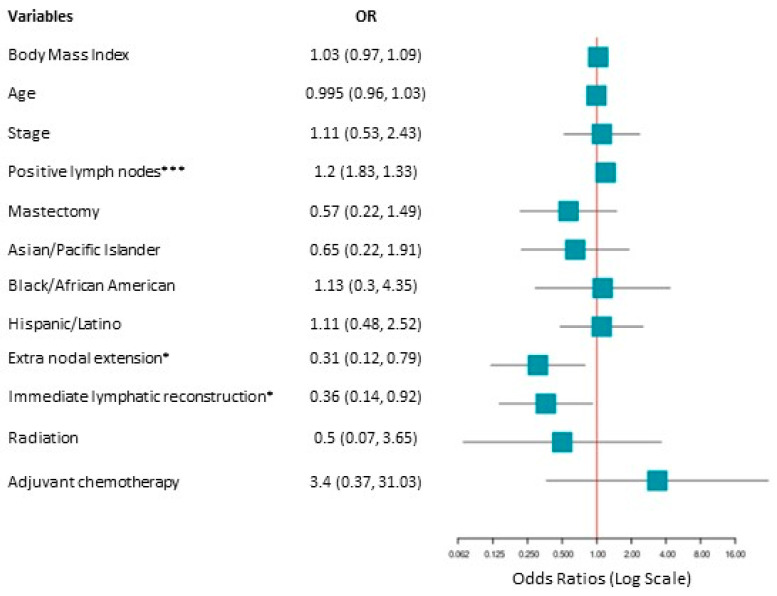
Odds ratio forest plot depicting demographic and clinical variables with the number of positive lymph nodes as a continuous variable. Body mass index and age were continuous variables. Stages compared stage 2 vs. stage 3: the reference category was stage 2. Positive lymph nodes were a continuous variable. Mastectomy: yes vs. no, with no as a reference category. Race/ethnicity: Non-Hispanic white was the reference category. Extranodal extension: no vs. yes, with no as the reference category. Immediate lymphatic reconstruction: no vs. yes, with no as the reference category. Radiation: no vs. yes, with no as the reference category. Adjuvant chemotherapy: no vs. yes, with no as the reference category. * *p* < 0.05; *** *p* < 0.001.

Due to the unequal group sizes, the regression model was reanalyzed with the amount of positive lymph nodes as a continuous variable. This model demonstrated three statistically significant factors associated with lymphedema development (Figure 3): the number of positive lymph nodes removed (*p* < 0.001), extranodal extension (*p* < 0.05), and ILR intervention (*p* < 0.05). The odds ratio for each positive lymph node removed was 1.2 (*p* < 0.001; 95% CI [1.08, 1.32]) suggesting that for each +LN removed there was a 20% increase in the odds of being diagnosed with lymphedema. The odds ratios for extranodal extension was 0.31 (*p* < 0.05; 95% CI [0.12, 0.79]) suggesting that those who had an extranodal extension diagnosis were about 70% less likely to have been diagnosed with lymphedema compared to those who had no evidence of extranodal extension. Similarly, the odds ratio for ILR was 0.36 (*p* < 0.05; 95% CI [0.14, 0.92]), which suggests that those who underwent ILR intervention were about 64% less likely to have a lymphedema diagnosis. The overall model for this analysis was also statistically significant (*X*^2^ (12, *N* = 186) = 25.96, *p* = 0.011).

Notably, the confidence interval is very wide, meaning that the odds ratio is statistically significant but not precise, so implications are made with a note of caution.

The onset of lymphedema developed later in those who received ILR compared to ALND only. Diagnosis was established at a mean post-operative day of 542 versus 389, respectively. The mean follow-up for patients who received ILR was 33 months compared to 39 months for controls. The mean time to end follow-up for both cohorts was at the three-year time point.

## 4. Discussion

Axillary lymph node excision is used for the diagnosis and treatment of breast cancer, with lymphedema being a common morbidity following surgery. Immediate lymphedema reconstruction is a microsurgical approach that maintains lymphatic flow by diverting it through the venous system. This analysis demonstrates that patients who received ILR during ALND experienced a lower overall rate of lymphedema compared to those who underwent ALND alone. Of those who underwent ILR and developed lymphedema, they went on to do so at a later date with a mean difference of 153 days. This suggests that ILR maintains continued flow and the delay of upper extremity lymphedema onset. These findings are similar to current literature [2,18].

ILR appeared to have a protective effect against lymphedema development. Only 18% of patients in the ILR group developed lymphedema compared to 31% of controls. Additionally, when all factors were held constant, undergoing ILR was shown to decrease the likelihood of developing postoperative lymphedema by 64%. A systematic review by Cook et al. [18] showed similar rates of control groups, but an even lower rate of development with ILR intervention, also in line with the current literature [12,19].

The strongest independent predictor of lymphedema development found in this analysis was the number of positive lymph nodes removed [12,19]. Groups who had 10–15 and 16+ +LNs removed had a 9 and 24 times increased likelihood of being diagnosed with lymphedema, respectively. For each +LN removed there was a 20% increase in the odds of being diagnosed with lymphedema. In contrast, this data found that extranodal extension was associated with a 70% decrease in diagnoses compared to patients who had no evidence of extension, but only when +LN was assessed as a continuous variable. The literature has shown that an increased number of positive lymph nodes is tied to advanced disease stage, yet data has not shown that late-staged tumors or clinically positive nodes are associated with lymphedema [2].

Age, race, and BMI were not factors shown to be significantly associated with lymphedema development for either control or interventional groups. Multiple studies have found no significant associations with age [20,21]. This data showed no correlation with race/ethnicity; however, Ren et al. [22] found that younger Black women experience an increased risk of lymphedema around 10 months after surgery compared to other age and race/ethnicity groupings. Kwan et al. [23] reported that African American patients faced nearly double the risk of developing BCRL compared with White/Caucasian patients. Increased BMI greater than 30 kg/m^2^ triples lymphedema risk [24] and obesity was found to be an independent risk factor for acquiring lymphedema [12,25]. Although this analysis did not reveal similar strong racial, age, or BMI associations, the current literature underscores the potential interplay of sociocultural factors, baseline comorbidities, and healthcare access in lymphedema risks warranting further investigation.

This analysis did not reveal any significant association between lymphedema development and radiation or chemotherapy despite a previously established increase [3,5]. Hara et al. reported a significant risk of lymphedema in patients receiving radiotherapy and, similarly, Taghian et al. noted that nodal irradiation and certain chemotherapy regimens may also contribute. A possible explanation for differing results may be due to the standardized regimen this cohort received, which could limit the capacity to detect differences. Chemotherapy, particularly neoadjuvant regimens, has been implicated in lymphedema [26,27,28], yet effects may have been diminished due to the protocol as well as the small sample size used in the analysis. Future investigations with more detailed data on individual chemotherapy agents, radiation techniques, and fractionation schedules could yield clearer insights into the relationship between these variables and BCRL.

ALND-only patients had a lymphedema rate of approximately 31%, aligning with prior studies citing 20–40% prevalence of postoperative lymphedema [4,5]. Although Kwan et al. observed a slightly lower incidence of 13.3% in their prospective cohort, differences in methodology (e.g., reliance on clinical coding rather than direct limb measurements) and demographic factors may partly account for the variation [26]. In contrast, other literature has shown a decreased lymphedema risk, which fell to 18% with prophylactic lymphatic reconstruction [13,15,29], while one small study failed to find a difference with or without ILR [29]. The ILR group took a longer time to obtain a lymphedema diagnosis, which may demonstrate that lymphatic channels were maintained and assisted with the delayed onset. However, differences in patient selection, surgical technique, and the definition of lymphedema may help to explain these inconsistencies.

Future studies can expand on this data through prospective studies with longer follow-ups and by continuing to use standardized lymphedema assessment tools (e.g., bioimpedance vs. circumferential measurements) to strengthen evidence. Additionally, a more in-depth analysis examining the cost-effectiveness [30] and impact on quality of life is warranted given that prior work suggests ILR may improve outcomes [31] and potentially reduce long-term healthcare costs [30].

### Limitations

This study had limitations inherent to its retrospective design and limited sample size. Subgroups had sparse or empty cells during data collection which violated the assumptions of Pearson’s chi-square test in categorical comparisons. We acknowledge that a non-parametric test could have been more appropriate, so all results were interpreted with caution. Using larger sample sizes in future studies will allow for more robust subgroup analyses in addition to using assumption-appropriate tests.

Menopausal status was recorded but not analyzed in this study. The established influence of hormonal receptor status on lymphedema requires additional assessment as a possible confounding variable and therefore was not assessed.

Discrepancies in this data may lie in patient demographics and sample sizes. Larger prospective trials that specifically stratify patients by menopausal status and age may help clarify whether and to what extent these factors truly affect BCRL risk.

## 5. Conclusions

Findings suggest that ILR at the time of ALND may both lower the incidence and delay the onset of lymphedema but does not prevent its development. The increased number of positive lymph nodes removed during axillary dissection were also noted to be significantly associated with the development of lymphedema. Age, menopausal status, BMI, race/ethnicity, radiation, and chemotherapy did not reach any statistical significance in the development of lymphedema.

## Figures and Tables

**Table 1:** Patient categorical information.

Category	Subcategory	ILR *n* = 44	Non-ILR *n* = 142
Age (years)		48 ± 11	55 ± 12
Menopausal status	Premenopausal	29	52
	Postmenopausal	15	90
Clinical tumor stage	cT1	4	21
	cT2	23	80
	cT3	12	26
	cT4	5	15
Clinical nodal stage	cN1	33	120
	cN2	5	16
	cN3	6	6
Histology	Invasive ductal carcinoma	40	126
	Invasive lobular carcinoma	4	16
Grade	1	0	5
	2	24	78
	3	20	59
Biomarkers	ER-positive HER2-negative	28	87
	HER2-positive	11	31
	Triple negative	5	24
Neoadjuvant chemotherapy		38	102
Initial axillary operation	Targeted axillary dissection	15	37
	Axillary lymph node dissection	29	105
Pathologic tumor stage	pT0	10	33
	pT1	16	47
	pT2	13	50
	pT3	5	10
	pT4	0	2
Pathologic nodal stage	pN0	12	31
	pN1	17	67
	pN2	13	31
	pN3	2	13
Residual cancer burden	Class 0	6	27
	Class I	2	9
	Class II	11	25
	Class III	15	33
Number of positive lymph nodes		4 ± 5	4 ± 3
Number of lymph nodes removed		16 ± 7	15 ± 6
Extranodal Extension		12	59
Adjuvant systemic therapy		44	134
Adjuvant radiation		42	139
Lymphedema		8	44
Distant recurrence		3	16
Mean follow-up (months)		33 ± 9	39 ± 5
Death		2	6

## Data Availability

The original contributions presented in this study are included in the article. Further inquiries can be directed to the corresponding author.
